# The Use of a Digitally Generated Matrix for Consistent Shade Recording in Tooth Bleaching—A Case Report

**DOI:** 10.3390/dj13080339

**Published:** 2025-07-24

**Authors:** Cristian Abad-Coronel, Guissell Vallejo-Yupa, Paulina Aliaga, Nancy Mena-Córdova, Jorge Alonso Pérez-Barquero, José Amengual-Lorenzo

**Affiliations:** 1 Digital Dentistry and CAD/CAM Materials Research Group, Faculty of Dentistry, Universidad de Cuenca, Cuenca 010107, Ecuador; 2 Department of Prosthodontics, Faculty of Dentistry, University of San Francisco de Quito, Quito 170901, Ecuador; gvallejo@estud.usfq.edu.ec (G.V.-Y.); nmena@usfq.edu.ec (N.M.-C.); 3 Faculty of Dentistry, Universidad San Francisco de Quito, Quito 170901, Ecuador; paliaga@usfq.edu.ec; 4 Department of Stomatology, Faculty of Medicine and Dentistry, University of Valencia, 46010 Valencia, Spain; jorge.alonso-perez@uv.es (J.A.P.-B.); jose.amengual@uv.es (J.A.-L.)

**Keywords:** shade guides, tooth bleaching, digital workflow, digital splints, color monitoring, spectrophotometer

## Abstract

**Objectives:** The aim of this study was to evaluate the effectiveness of spectrophotometers for objective tooth color measurement, particularly in bleaching procedures enhanced by digital positioning templates. **Methods:** Tooth color registration was conducted using both subjective methods with shade guides and objective methods with spectrophotometers. Spectrophotometers were chosen for their ability to provide objective, quantifiable, and reproducible results, crucial for monitoring color modifications accurately. Digital workflows were implemented to enhance the registration process further. These workflows included providing a precise positioning matrix for spectrophotometer sensors and optimizing working models to ensure high-quality therapeutic splints. **Results**: The use of spectrophotometers demonstrated superior performance in registering tooth color objectively compared to subjective shade guides. Digital workflows significantly improved the precision and efficiency of spectrophotometer measurements through a digital matrix, enhancing the quality of therapeutic splints obtained. **Conclusions**: Spectrophotometers are recommended for objective and precise tooth color registration, particularly in bleaching procedures. Integrating a digital positioning matrix enhances measurement accuracy and reliability, supporting effective monitoring and treatment outcomes.

## 1. Introduction

The registration of tooth color occurs when light of a specific wavelength is reflected from a tooth and corresponds to the color perceived by an observer’s brain or a measurement instrument, such as a spectrophotometer, after analyzing the reflected light. This is a complex procedure that requires adequate observer capability, theoretical knowledge, training in the scientific principles governing shade evaluation, and specific instruments and technologies for this purpose [1].

In dentistry, tooth color can be determined in two ways: subjectively, using dental shade guides, and objectively, using instruments specifically designed for this purpose [1,2,3,4]. In the subjective method, the observer visually compares shade tabs with the patient’s teeth and selects the closest match. However, this method lacks reproducibility and is sensitive to external factors such as lighting conditions [5]. By contrast, objective methods employ spectrophotometers, which integrate their own calibrated light source and are capable of highly reproducible measurements.

In the context of tooth bleaching, evaluating the progressive color change presents additional challenges. This evaluation requires multiple measurements over time to monitor changes and determine when the tooth color has stabilized. Quantitative methods that assign numerical values to color differences are therefore preferable. Subjective methods do not allow the numerical expression of color changes, whereas objective instruments quantify these changes by translating color parameters (hue, saturation, and brightness) into numerical data that can be analyzed and compared at each treatment stage.

Currently, instruments and technologies for dental shade measurement, including colorimeters, spectrophotometers, spectroradiometers, and digital color analyzers, present limitations such as a higher cost compared to visual shade guides, the need for specialized knowledge [6,7,8], and the difficulty of ensuring consistent positioning on the curved surfaces of teeth. A key gap identified in the literature is the absence of standardized protocols to ensure consistent spectrophotometer positioning on tooth surfaces in real clinical scenarios, which can significantly affect measurement reproducibility and reliability. Nevertheless, they remain essential for objectively quantifying color change during bleaching treatments.

To overcome these positioning challenges, the development of a digitally designed positioning matrix specifically adapted for spectrophotometers has been proposed. This matrix ensures the accurate and repeatable placement of the instrument at the same location on the tooth surface, minimizing errors due to variations in positioning and tooth morphology. Furthermore, the integration of digitally fabricated splints, tailored through advanced CAD/CAM workflows to fit both the patient’s dentition and the spectrophotometer tip, offers additional advantages: enhanced reproducibility, reduced chairside time, lower costs, and improved patient comfort.

By stabilizing the spectrophotometer and reducing operator-related variability, these advancements improve both the accuracy and reliability of dental color measurements, contributing to more predictable clinical outcomes. Recent studies have shown that such positioning systems significantly improve the consistency of esthetic evaluations, supporting their potential for widespread adoption in clinical practice and research [9,10,11,12].

This study presents a case of combined vital tooth bleaching in which color changes were quantified using a spectrophotometer in conjunction with a rigid, custom-fabricated positioning matrix designed and produced through digital workflows.

The experimental approach described in this report addresses a critical gap in the reproducibility of dental color evaluations. By ensuring the standardized positioning of the spectrophotometer, the proposed methodology minimizes operator variability and mitigates errors due to tooth convexity and other anatomical factors. These improvements are particularly relevant given the documented challenges in achieving reliable shade measurements, as demonstrated in comparisons between freehand techniques and standardized protocols using commercial instruments such as the VITA Easyshade and SpectroShade [10].

Moreover, this approach aligns with research emphasizing the importance of precise instrument positioning for consistent ΔE values, a key metric in dental color science. Studies have shown that standardizing the instrument positioning reduces inter-operator variability and improves repeatability across different clinical settings. These innovations not only enhance clinical efficiency but also raise the standard of esthetic evaluations, positively influencing patient satisfaction and treatment predictability. Incorporating this protocol into routine clinical workflows has the potential to substantially improve the accuracy and reliability of dental shade assessments, reinforcing its relevance for both clinical and research applications [10].

## 2. Materials and Methods

The patient treated was a 25-year-old male presenting with constitutional discoloration aggravated by inadequate hygiene and dietary habits. Initially, a medical history was taken, and a clinical and radiographic examination was performed. After evaluating the type and degree of discoloration, a combined vital bleaching treatment was planned for his central and lateral incisors, canines, and premolars of both arches, consisting of an initial in-office phase followed by an at-home phase using individualized trays.

The patient was informed about the treatment characteristics, requirements, and potential risks [10,11] and signed an informed consent. The patient was treated under the authorization of the Human Research Ethics Committee of the Experimental Research Ethics Committee of the University of Valencia, with registration number 1234846.

Both arches of the patient were scanned using a blue-light, ultra-high-resolution scanner (PrimeScan, Dentsply Sirona, Germany). The obtained STL files were exported via a dedicated platform (DS Connect). Using laboratory design software (InLab 22.0, Dentsply Sirona, Germany), a positioning matrix with spectrophotometer slots was designed on the scanned model to consistently position the sensor on the same area of the index teeth to be evaluated (upper central incisors and upper and lower canines) with a tolerance spacer of 100 microns. Additionally, reservoirs for the bleaching agent were designed for the therapeutic trays.

**Figure 1:**
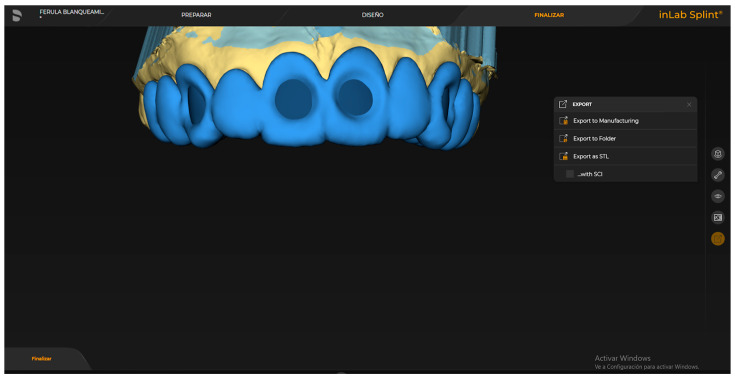
Digitally designed positioning matrix showing the slot for precise and reproducible placement of the spectrophotometer sensor on the buccal surface of the index teeth.

The positioning matrices were designed with a thickness of 2 mm, extending to the second premolars, with a rounded hole in the center of the buccal surface 1 mm larger than the diameter of the spectrophotometer sensor to be used (6 mm) (Figure 1). The proper positioning of the spectrophotometer (Vita, Bad Säckingen, Germany) is crucial for obtaining accurate results in dental shade measurement. Moving the instrument to different areas of the tooth or altering its angle can result in significant variations in measurements due to differences in translucency, reflection, and chromaticity across dental zones. Furthermore, changes in the distance between the spectrophotometer and the tooth surface can also affect the recorded color parameters. Therefore, the instrument must remain stable, perpendicular to the tooth, and properly calibrated to ensure consistent conditions and minimize errors in shade selection [12].

Reservoirs were designed on each tooth to be bleached, both on the buccal and lingual/palatal surfaces, as thin layers with a thickness of 0.4 mm, located 3 mm from the incisal edge and the cemento–enamel junction, and 2 mm from the mesial and distal surfaces.

**Figure 2:**
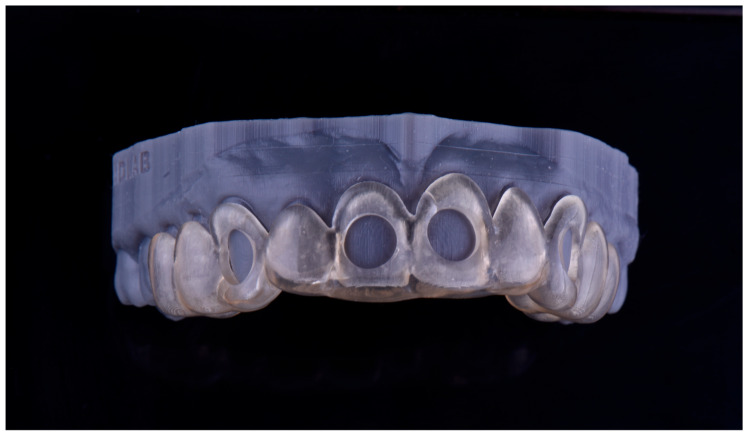
3D-printed positioning matrix fabricated in resin, designed to ensure precise and repeatable placement of the spectrophotometer on the tooth surface.

The positioning matrix and working models with integrated reservoirs were designed using CAD software (InLab 22, Dentsply-Sirona, Bensheim, Germany), applying a 100 μm tolerance spacer to ensure a passive and precise fit. These components were printed using a digital light processing (DLP) 3D printer (Sprintray Pro 95, SprintRay, Los Angeles, CA, USA) with a splint-specific resin (Splint, SprintRay, CA, USA) (Figure 2). The printing resolution was set at 50 μm layer thickness and 50 μm XY resolution, ensuring the high fidelity and dimensional accuracy of the final matrices.

The bleaching trays were fabricated on the printed working models with incorporated reservoirs. Soft plastic sheets (1.5 mm thickness) (Soft-Tray Sheets 1.5 mm, Ultradent Products Inc., South Jordan, UT, USA) were used and cut to follow the gingival line without covering the gums [13,14].

Before starting the in-office phase, plaque and stains on the external tooth surfaces were removed using a non-abrasive cleaning paste applied with a nylon brush mounted on a low-speed contra-angle.

**Figure 3:**
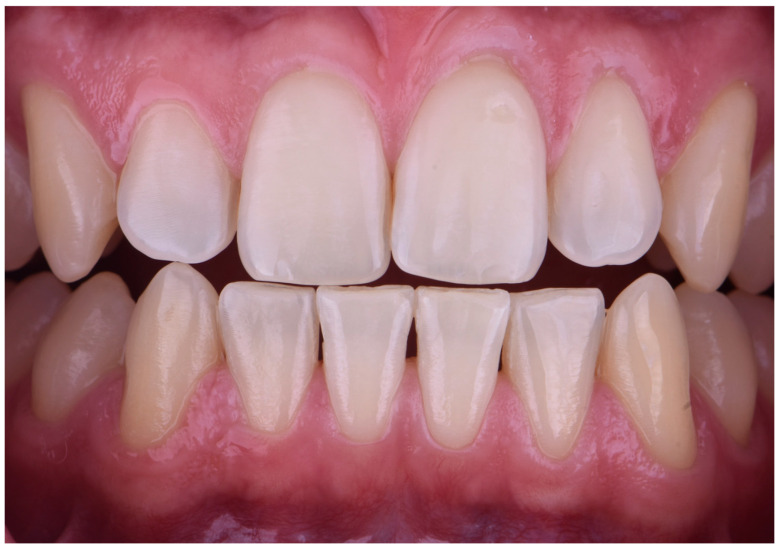
Initial intraoral digital photograph showing baseline tooth shade.

Intraoral digital photographs were taken: one in an edge-to-edge position of the central incisors and another with the Bleachedguide (Vita, Bad Säckingen, Germany) color reference closest to the untreated tooth color, placed on the same axis and plane in an edge-to-edge position with the right maxillary central incisor [15] (Figure 3).

**Figure 4:**
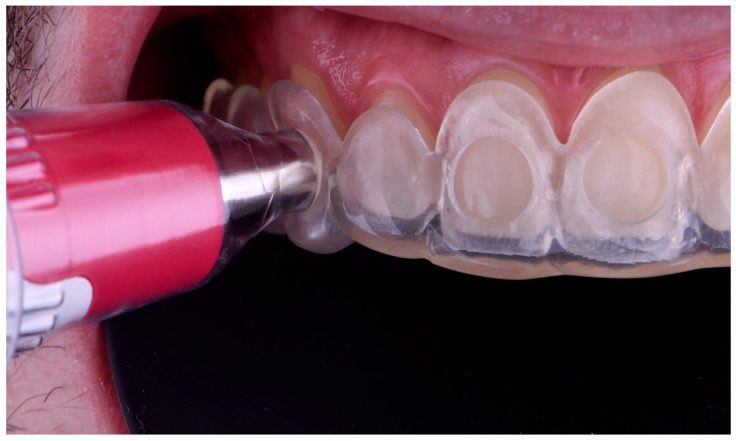
Color measurement procedure using the spectrophotometer positioned on the digitally fabricated positioning matrix to ensure reproducibility.

The first color measurement was then performed with the spectrophotometer, recording the CIELab color space parameters L * (lightness), a * (red–green saturation variation), and b * (yellow–blue saturation variation) of the index teeth using the Easyshade V spectrophotometer [16,17] (Figure 4).

**Figure 5:**
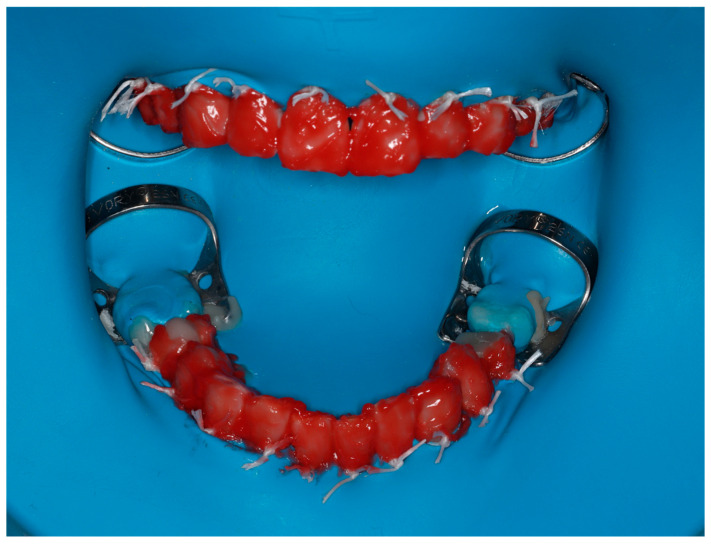
In-office bleaching phase showing isolation with rubber dam and application of 40% hydrogen peroxide gel on buccal and palatal/lingual tooth surfaces.

The in-office phase was performed by isolating the teeth with a double-arch rubber dam (OptiDam, Kerr, Bioggio, Switzerland) inverted into the gingival sulci and sealed with silk ligatures around the necks of each isolated tooth [18]. A 40% hydrogen peroxide product (Opalescence™ Boost™, Ultradent Products Inc., South Jordan, UT, USA) in a dual syringe was applied, which, after mixing, forms a red gel (Figure 5). Two 20 min applications were performed (as recommended by the manufacturer) on both the buccal and palatal/lingual surfaces due to the use of the rubber dam [19].

After each application, the product was aspirated with a capless ejector, the remaining product was rinsed off with water, and the teeth were dried with air. After the second application, the cervical ligatures and rubber dam were removed, a second color measurement was taken, and the color differences (ΔE1) between the initial and post-in-office phase color parameters were calculated [20].

Finally, the patient was instructed to maintain routine oral hygiene, avoid smoking, and refrain from consuming pigmented foods and beverages until the start of the at-home phase, throughout the at-home phase, and for at least one week after its completion [21,22].

Seven days after the in-office phase, the patient was reviewed to assess the oral tissue condition and absence of complications, and a third color measurement (ΔE2) was taken. The patient was shown how to load the trays with the at-home bleaching agent and apply them in the mouth. He was provided sufficient product for one week (three tubes) of a 16% carbamide peroxide gel (Opalescence™ PF, Ultradent Products Inc., South Jordan, UT, USA) in a syringe with an applicator tip, along with detailed instructions for use according to the planned therapeutic regimen (90 min per day) [23].

Weekly reviews were conducted to assess oral tissues and monitor for complications, with color measurements and ΔE calculations performed until ΔE values stabilized without variation (ΔE4, ΔE5, and ΔE6).

**Figure 6:**
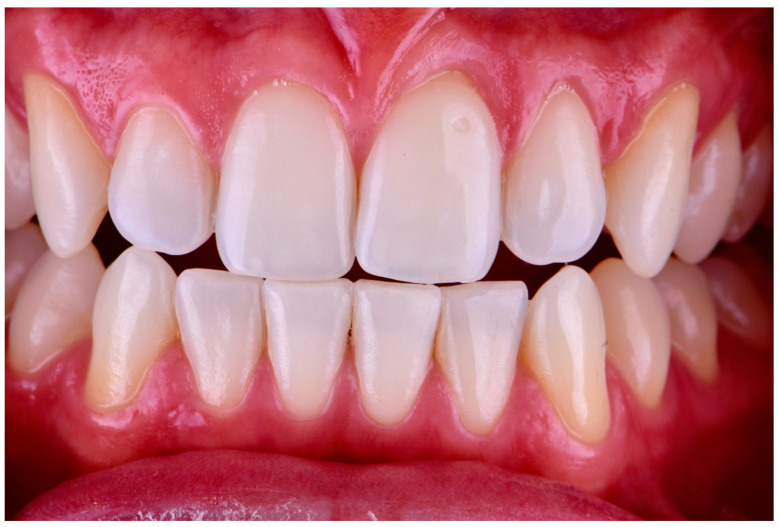
Final intraoral view showing post-treatment results and color stability one week after completion of the at-home bleaching phase.

One week after achieving color stability, the photographic records described above were repeated, and the ΔE7 was calculated one week after completion of the at-home phase [11] (Figure 6).

All color measurements and photographic records were performed by the same calibrated operator to minimize the risk of bias in data collection. A perceptibility threshold of ΔE > 2.7 units was employed to consider the color changes achieved at the conclusion of the combined bleaching treatment as clinically acceptable [10].

## 3. Results

Three tables are presented showing the ΔE values obtained for the evaluated teeth following the in-office session (ΔE1) and during the follow-up appointments after 5 and 7 weeks of at-home treatment (ΔE5 and ΔE6), during which the ΔE values stabilized (Table 1, Table 2 and Table 3).

## 4. Discussion

In the last decade, research has primarily focused on the digital planning of guided treatments to improve precision and predictability in complex dental procedures, such as orthognathic surgery, implant planning and placement, orthodontic therapy, complex endodontic treatments, and particularly in prosthodontics [24]. Therefore, the importance of adopting these tools today lies in supporting clinicians in procedures commonly performed in daily dental practice—not necessarily complex ones—such as dental bleaching.

To enhance the precision and accuracy of workflows across all areas of dentistry, computer-aided design (CAD) and computer-aided manufacturing (CAM) technologies have been increasingly utilized. Within this context, virtual treatment planning enables the prediction of favorable outcomes, with a particular emphasis on the fabrication of computer-designed and manufactured trays and matrices aimed at guiding the spectrophotometer sensor’s positioning on the tooth surface [25].

In addition to focusing solely on color change, it is important to recognize that bleaching procedures may influence subsequent clinical outcomes, such as the adhesion of orthodontic brackets or restorative materials. Therefore, ensuring an accurate color assessment and allowing sufficient time for color stabilization are essential considerations before proceeding with further treatments. This broader perspective underscores the need for comprehensive planning and highlights the role of digital workflows in enhancing clinical precision. Employing a digital workflow in bleaching offers advantages such as maintaining the dimensional stability of working models and preventing common issues associated with traditional techniques, including impression-taking errors, casting inaccuracies, fractures of stone models during trimming, and poorly designed or improperly positioned reservoirs. Additionally, it facilitates the creation of positioning matrices that allow for greater reproducibility when positioning the spectrophotometer sensor on the tooth surface [26].

The resin used is biocompatible and specifically formulated for occlusal splints, offering superior flexural strength compared to other materials employed for similar purposes, such as rigid acrylic, flexible materials like polyurethane or silicones, thermoplastics like ethylene-vinyl acetate, or the more commonly used polycarbonate. Its flexural strength and mechanical properties enable reliable results with fewer variables, minimizing errors during information acquisition [27].

A spectrophotometer was selected for the tooth color measurement because it is an objective method with high reproducibility, independent of ambient light conditions or operator skill. It allows for the numerical quantification of color changes [28].

In this case report, the spectrophotometer sensor was consistently positioned on the same area of the labial surface of the evaluated teeth for each measurement by means of the positioning matrix, ensuring that all readings were taken under identical conditions. This approach eliminates minor positional deviations that could otherwise alter results [29]. This digital colorimetric tool, based on the CIELab color system, is recognized as an objective and quantitative method for evaluating color changes in vital teeth, capable of detecting subtle variations in localized areas that are difficult to perceive visually [30].

The calculation of ΔE is considered an appropriate method for quantifying and monitoring the color changes produced by dental bleaching, as it yields a numerical value derived from the initial L*, a*, and b* parameters of the tooth or reference, compared to those recorded at various stages of the bleaching process. This value represents the Euclidean chromatic distance between the pre-bleaching color and that observed at subsequent treatment stages, calculated using the formula [31]: ΔE = [(Lf − Li)^2^ + (af − ai)^2^ + (bf − bi)^2^]½.

During the clinical bleaching phase, rubber dam isolation and cervical floss ligatures were employed to prevent the bleaching agent from contacting the oral soft tissues, thereby preserving their integrity [32].

The at-home bleaching trays were designed with small-capacity reservoirs to facilitate the application and retention of the bleaching agent, preventing overflow and subsequent contact with the oral soft tissues.

The bleaching treatment was concluded once very similar ΔE values were obtained in two consecutive measurements during the at-home phase (ΔE5 and ΔE6), indicating that tooth color stabilization had been achieved. Achieving color stabilization helps prevent or delay discoloration relapse following bleaching.

Only by objectively quantifying the color of bleached teeth can clinicians accurately evaluate the degree of color change and determine the point at which further whitening ceases to occur. Thus, integrating a digital workflow into dental bleaching optimizes the recording of tooth color by standardizing the positioning of measuring instruments, ensuring that objective, reproducible, and consistent numerical records are obtained. These records enable clinicians to determine when a tooth has reached its maximum achievable whitening. Uncontrolled movements during the measurement can lead to significant errors, particularly due to variable tooth translucency and differences across dental surfaces. By maintaining the spectrophotometer in a constant, repeatable position, clinicians can obtain accurate and reproducible records that are essential for a precise dental color assessment, especially in bleaching procedures. In this way, the positioning matrix not only ensures measurement precision but also facilitates the consistent comparison and monitoring of results over time.

However, incorporating digitally designed and manufactured positioning splints into routine clinical practice requires the availability of a minimum technological infrastructure—such as that analyzed in this study—as well as adequate training to enable their correct design, fabrication, and clinical use.

## 5. Conclusions

The use of a digital workflow enabled the creation of working models for fabricating custom trays used by the patient for the at-home application of the bleaching agent.The incorporation of a digitally designed positioning matrix in dental color measurements with a spectrophotometer is essential for achieving precise and consistent results. This matrix ensures that the instrument remains in a stable and standardized position, maintaining correct angulation between the sensor and the tooth, and preventing movements that could alter color parameters due to variations in distance, angle, or the specific area being measured.

Further studies involving larger sample sizes and comparisons with other color measurement instruments that can be positioned using digitally fabricated splints are recommended to determine the true efficacy and clinical relevance of this approach.

## Figures and Tables

**Table 1:** ΔE values obtained from the evaluated teeth after the in-office session.

	L. Initial	L. Initial	a. Final	a. Initial	b. Final	b. Initial	∆E1
**1.3**	77.70	79.70	1.70	1.20	29.00	28.90	2.06
**1.1**	79.90	83.90	−0.90	−1.70	17.50	16.20	4.28
**2.1**	80.40	85.40	0.00	−1.50	20.10	17.50	5.83
**2.3**	77.60	80.00	2.20	1.60	29.80	29.60	2.48
**3.3**	80.90	79.10	1.50	1.00	28.90	26.90	2.74
**4.3**	79.10	81.40	1.80	0.70	28.70	26.80	3.18

**Table 2:** ΔE values obtained after 5 weeks.

	L. Final	L. Initial	a. Final	a. Initial	b. Final	b. Initial	∆E5
**1.3**	88.30	79.70	−2.20	1.20	12.40	28.90	18.91
**1.1**	88.80	83.90	−2.40	−1.70	7.60	16.20	9.92
**2.1**	87.40	85.40	−2.50	−1.50	6.70	17.50	11.03
**2.3**	88.40	80.00	−2.20	1.60	13.20	29.60	18.81
**3.3**	88.50	79.10	2.00	1.00	14.70	26.90	15.43
**4.3**	88.60	81.40	−2.30	0.70	13.80	26.80	15.16

**Table 3:** ΔE values obtained after 7 weeks.

	L. Final	L. Initial	a. Final	a. Initial	b. Final	b. Initial	∆E6
**1.3**	88.30	79.70	−2.20	1.20	12.20	28.90	19.09
**1.1**	88.80	83.90	−2.40	−1.70	7.80	16.20	9.75
**2.1**	87.40	85.40	−2.50	−1.50	6.90	17.50	10.83
**2.3**	88.40	80.00	−2.20	1.60	13.00	29.60	18.99
**3.3**	88.50	79.10	2.00	1.00	14.40	26.90	15.67
**4.3**	88.60	81.40	−2.30	0.70	14.00	26.80	14.99

## Data Availability

No new data were created or analyzed in this study. The data supporting the reported results are publicly available.
